# Neurophysiological Measures and Alcohol Use Disorder (AUD): Hypothesizing Links between Clinical Severity Index and Molecular Neurobiological Patterns

**DOI:** 10.4172/2155-6105.1000181

**Published:** 2016-04-26

**Authors:** Mario Vitali, Carmen Napolitano, Marlene Oscar Berman, Simona Flamminii Minuto, Gemma Battagliese, Maria Luisa Attilia, Eric R Braverman, Marina Romeo, Kenneth Blum, Mauro Ceccanti

**Affiliations:** 1 Alcohol Addiction Program Latium Region Referral Center, Sapienza University of Rome; 2 Department of Psychiatry and Neurology, Boston University School of Medicine and Veterans Administration System, Boston, Massachusetts, USA; 3 Department of Psychiatry & McKnight Brain Institute, University of Florida, College of Medicine, Gainesville, Florida, USA; 4 Department of Clinical Neurology, Path Foundation, NY, New York, New York, USA; 5 Department of Addiction Research & Therapy, Malibu Beach Recovery Center, Malibu Beach, California, USA; 6 Department of Psychiatry & Human Integrated Services Unit University of Vermont Center for Clinical & Translational Science, College of Medicine, Burlington, Vermont, USA; 7 Department of Nutrigenomics, RD Solutions, LLC, La Jolla, California, USA; 8 Department of Personalized Medicine, IGENE, LLC, Austin, Texas, USA; 9 Dominion Diagnostics, LLC, North Kingstown, Rhode Island, USA; 10 Basic & Clinical Research Center, Victory Nutrition, LLC., Austin, Texas, USA

**Keywords:** TCI, Addiction, Craving, Reward Deficiency Syndrome (RDS), Alcohol Dependence (ADD)

## Abstract

**Background:**

In 1987, Cloninger proposed a clinical description and classification of different personality traits genetically defined and independent from each other. Moreover, he elaborated a specific test the TCI to investigate these traits/states. The study of craving in Alcohol Use Disorder (AUD) assumed a greater significance, since ever more data seems to suggest a direct correlation between high levels of craving and a higher risk of relapse in alcoholics. Thus, our study aim is to explore the possible correlations among TCI linked molecular neurobiological pattern (s), craving and alcohol addiction severity measures in a sample of Italian alcoholics.

**Materials and Methods:**

191 alcoholics were recruited in a Day Hospital (DH) setting at the Alcohol Addiction Program Latium Region Referral Center, Sapienza University of Rome. After 7 days detoxification treatment a psychodiagnostic protocol was administered, including TCI, VAS-C, ASI and SADQ. All patients signed an Institutional Review Board (IRB) approved informed consent.

**Results:**

Principally, we detected a significant positive correlation between HA-scale scores and the VAS scale: increasing in HA-scale corresponds to an increase in craving perception for both intensity (r=0.310; p ≤ 0.001) and frequency (r=0.246; p ≤ 0.001). Moreover, perception of dependence severity, measured with SADQ was also found to be significantly associated positively to both HA-scale (r=0.246; p ≤ 0.001) and NS-scale (r=0.224; p ≤ 0.01). While, for character scales, Persistence (r=−0.195; p=.008) and Self-directedness (r=−0.294; p ≤ 0.001) was negatively associated with ASI linked to alcohol problems. Self-directedness was also negatively correlated with ASI linked to family and social problems (r=−0.349; p ≤ 0.001), employment and support problems (r=−0.220; p=0.003) and psychiatric problems (r=−0.358; p ≤ 0.001). Cooperativeness was a negative correlate with Legal Problems (r=−0.173; p=0.019). and Self-Transcendence was positive correlated with Medical Problems (r=0.276; p ≤ 0.001)

**Conclusions:**

In view of recent addiction neurobiological theories, such as the “Reward Deficiency Syndrome (RDS)” and the Koob model, our data could suggest that our cohort of patients could possibly be in a particular stage of the course of their addiction history. Thus, if our hypothesis will be confirmed, the TCI-based assessment of alcoholics would allow an optimization of the treatment. Clinicians understanding these newer concepts will be able to translate this information to their patients and potentially enhance clinical outcome (s), because it could suggest a functional hypothesis of neurotransmitter circuits that helps to frame the patient in his/her history of addiction.

## Introduction

In 1987, Cloninger proposed a systematic method for the clinical description and classification of different personality traits, based on the Biosocial Theory [1]. This theory is based on several neuropharmacological, neuroanatomical, neurophysiological and psychometric studies conducted on personality structure, that hypothesize a partition of temperament in three dimensions, genetically defined and independent from each other, respectively associated to the function of three neurotransmitters. These dimensions interact together in adaptive responses to specific environmental stimuli, building typical personality traits/states [2,3]. These three temperament dimensions, described by Cloninger, are: Novelty Seeking, Harm Avoidance and Reward Dependence. The first one (NS – Novelty Seeking) notices the propensity to pronounced excitement in response to new stimuli; the second one (HA – Harm Avoidance) describes the propensity to avoid aversive stimuli acting strategies in order to prevent punishments. The last dimension (RD-Reward Dependence) related to impairments of the “brain reward cascade” leading to reward deficiency syndrome (RDS) and indicates the subject’s predominant reliance on external gratification, with a strong tendency of keeping positively supported behaviors and avoiding punishments. In the original view the NS is inversely related to the dopamine system, the RD is inversely related to the noradrenergic system while the HA is positively related with the serotonin system. However, with an emerging fields of Psychiatric Genetics and Genomics multiple genes are involved in all these behaviors and it is more complex [4].

Cloninger [1] has linked the combinations of these three temperamental index to several Personality Disorders (PD) described in DSM IV [5]. Moreover, Cloninger [1] developed a specific test that investigates those three described areas and four other factors (Persistence, Self-directedness, Cooperativeness, Self-Transcendence), non-correlated with specific neurotransmitters, but indicating some other behavioral peculiarities. Cloninger designed a self-report questionnaire aimed to identify specific temperamental and behavioral patterns of the distinct population of patients. This tool, the *Temperament and Character Inventory (TCI)*, in its original version 9, was composed by 240 items on a dichotomous scale (true–false) [6].

TCI was used in several pathological populations, including subjects with drugs and alcohol addiction [7,8]. Several studies have been published on the relationship between a number of particular personality traits and different types of alcoholism [8,9]. Cloninger proposed two types of alcoholism with distinctive clinical features and TCI profiles.

The Type I describes a kind of alcoholism with an onset of addiction over the age of 25, without familiarity or any important comorbidities. These patients score high on the HA and RD subset of the TCI.

The Type II, clinically presents an early-onset of addiction patterns, with important familiarity and comorbidities (for example cluster B personality disorders, bipolar disorders etc.). Moreover, psychosocial problems could be associated these patients score high on both NS and HA subset of the TCI [9–22].

## Craving in Alcohol Use Disorder (AUD)

“Craving” is a clinical concept, ambiguously definable. It can be explained as a set of cognitive, behavioral and physiological symptoms indicating the intense and uncontrollable desire of consuming a psychotropic substance, the effects of which have already been experienced [23]. It is well established that craving can be a strong predictor of relapse [24,25], and in the current classification of mental disorders (DSM-5), craving is one of the criteria for the diagnosis of addiction [26]. In substance abuse, craving is characterized by the presence of some key features, such as the strong attraction (impulsive and/or compulsive) to situations that allow the intake of substances and the behavioral activation, cognitive and emotive, featured by multiple sets of symptoms. Anton et al. [27] suggested that many traits of alcohol craving are similar to some clinical features of OCD (Obsessive – Compulsive Disorder) patients. They point out that craving may be linked to recurrent and persistent thoughts about alcohol; the subject’s inability to resist these thoughts; compulsive thrust to alcohol-intake; and loss of control [27]. Even if alcohol craving was traditionally conceptualized as a singular-dimensional condition [28], the modern view as espoused by the American Society of Addiction Medicine (ASAM) recently defined addiction as a complex brain disorder involving RDS [29,30] due to reward circuitry impairments.

Moreover, contemporary theorists recognize nowadays a dynamic competition between the tendencies to approach drinking and the ones to avoid drinking [31]. Meanwhile, there was a growing emphasis about the distinction between a) craving as “entity”, i.e. the simple, unidirectional desire of drinking and b) this desire seen as a “process”, or a more complex experience that requires consideration of many contextual factors. Finally, there is a strong impetus to integrate the complexities of both “molecular neurogenetics” and “psychological processes” that seems to be important antecedents to substance related seeking behavior (e.g. craving) [32]. McEvoy et al. [33] compared three models of craving, which reflect the evolution of the theory from a) traditional uni-dimensional model to b) bi-dimensional model of “ambivalence [31].” and after all to c) a “neuroanatomical” model that adheres to the dimension of avoiding, and as such integrates it with the multiple brain processes involved in reward, obsessive-compulsive behaviors and inhibitory processes [33–35].

In terms of Alcohol Use Disorder (AUD) the singular concept of just craving as the reason for relapse takes on a more complex dynamic when one compares our earlier non-genetic view with our modern view. A brief review of the current literature would suggest that a more parsonomiuos view is that relapse is due to a direct correlation between high levels of craving and a higher risk of relapse in alcoholics [36–42] including dopaminergic genetic antecedents including epigenetics [43].

Thus, the aim of the present study is to investigate the possible correlations between TCI “phenomena” and putative molecular neurobiological antecedents, substance –related seeking and substance (*i.e.* alcohol) addiction severity indices in a sample of Italian alcoholics.

## Materials and Methods

### Subjects

The sample investigated consisted of 191 subjects (male n=133, female n=58; male average-age: 42.77 ± 9; female average-age: 41.78 ± 7). Patients were eligible according to the following inclusion/exclusion criteria. **Inclusion Criteria:** 1) Signed informed consent form; 2) Age between 18 and 65 years old; 3) Italian native-speakers; 4) Diagnosis of alcohol abuse/dependence according DSM IV TR criteria; 5) Hospitalization in Day Hospital; 6) At least 7 days of alcohol abstinence. **Exclusion Criteria:** 1) Pregnant or lactating women; 2) Severe renal failure; 3) Decompensated liver cirrhosis; 4) Positive anamnesis for Deliberate Self-Harm (DSH); 5) Epilepsy; 6) Unstable or severe heart diseases; 7) Neoplastic Diseases.

All selected patients received a detoxification and rehabilitation integrated treatment in a hospitalization setting (Day Hospital, DH) at the Alcohol Addiction Program Latium Region Referrral Center, Sapienza University of Rome. The study was approved by the hospital’s IRB committee as well as the informed consent was signed by each participant.

### Study Protocol

The three study phases (alcohol detoxification, screening, psychodiagnostic assessment) are summarized in Table 1.

#### Phase 1: Alcohol detoxification

During the first 7 days of the study alcohol detoxification was performed in the DH setting according to the ‘symptom-triggered’ therapy. This therapy consists of monitoring patients and providing medication only when symptoms of alcohol withdrawal appear. The use of a simple, objective and standardized scale (Clinical Institute Withdrawal Assessment for Alcohol, revised Scale, CIWA) by the clinicians appears safe and effective in monitoring the patient’s clinical conditions. Usual prescriptions are for diazepam 20 mg orally (or chlordiazepoxide 100 mg orally) as needed hourly for a CIWA-Ar score of >10. Nurses scored the patients at hourly intervals in early withdrawal. Drug doses are repeated until the appropriate therapeutic responses (suppression of symptoms of withdrawal) occurred [44].

#### Phase 2 Screening

Screening was performed at day 8 of the study according to the inclusion/exclusion criteria listed in Table 1.

#### Phase 3: Psycho-diagnostic assessment

Psycho-diagnostic assessment was performed between day 9 and 13 of the study to explore alcohol-related, cognitive and character dimensions of the subjects (Table 1)

#### AUD assessment

The ASI (Addiction Severity Index fifth edition) is a semi-structured weighted interview built with the purpose of gathering information about the patient’s life [45,46].

The Severity of Alcohol Dependence Questionnaire (SADQ) is a tool used to analyze the severity of the addiction through a series of symptoms referred to the maximum alcohol-abuse period [47,48]. The SADQ consist of a self-report questionnaire of 20 items with 0 to 3 scores; this test evaluates the perceived frequency of physical and psychological symptoms of alcohol abuse. It is composed by a set of questions about some aspects of the dependency syndrome: withdrawal symptoms, affective symptoms, frequency of alcohol intake, and time to the onset of withdrawal symptoms.

The Visual Analogue Scale – Craving (VAS –C) uses a line of 10 cm to measure the intensity and the frequency of the perceived craving for alcohol during the week before the admission in DH to Alcohol Addiction Program Latium Region Referrral Center, Sapienza University of Rome. In order to rate the intensity of the craving, subjects are asked to mark, on a scale of 1 to 10, whereby 1 indicate low desire and 10 indicates high desire to drink. For the frequency, subjects indicate how many times a day they feel their need to drink on a scale of 1 to 10 as well [49].

#### Cognitive assessment

The Mini Mental State Examination (MMSE) is an early screening test designed to detect early cognitive impairment, which has been correlated with electrophysiological measures to predict severity [50,51].

The Wechsler Adult Intelligent Scale (WAIS -R battery) provides information regarding the presence of learning difficulties due to previous to the alcohol abuse [52,53].

#### Temperament and character assessment

To assess personality traits (temperament and character) we used the version 9 of TCI test [22] (Italian translation by Battaglia and colleagues) [54].

## Data Analysis

Statistical analyses described in this section were performed by SPSS version 13 for Windows package. A preliminary inspection of the distributional properties of the measures was conducted. Normality was tested on the outcome variables which were considered normally distributed. If skewness and kurtosis were found these variables were transformed with a logarithmic transformation. Pearson correlation coefficients were computed for evaluating relationships across the continuous variables considered. For the TCI scales the raw scores were used. In order to control Type1 error, Bonferroni correction was used and the significance level was set at .025.

## Results

**Figure 1:**
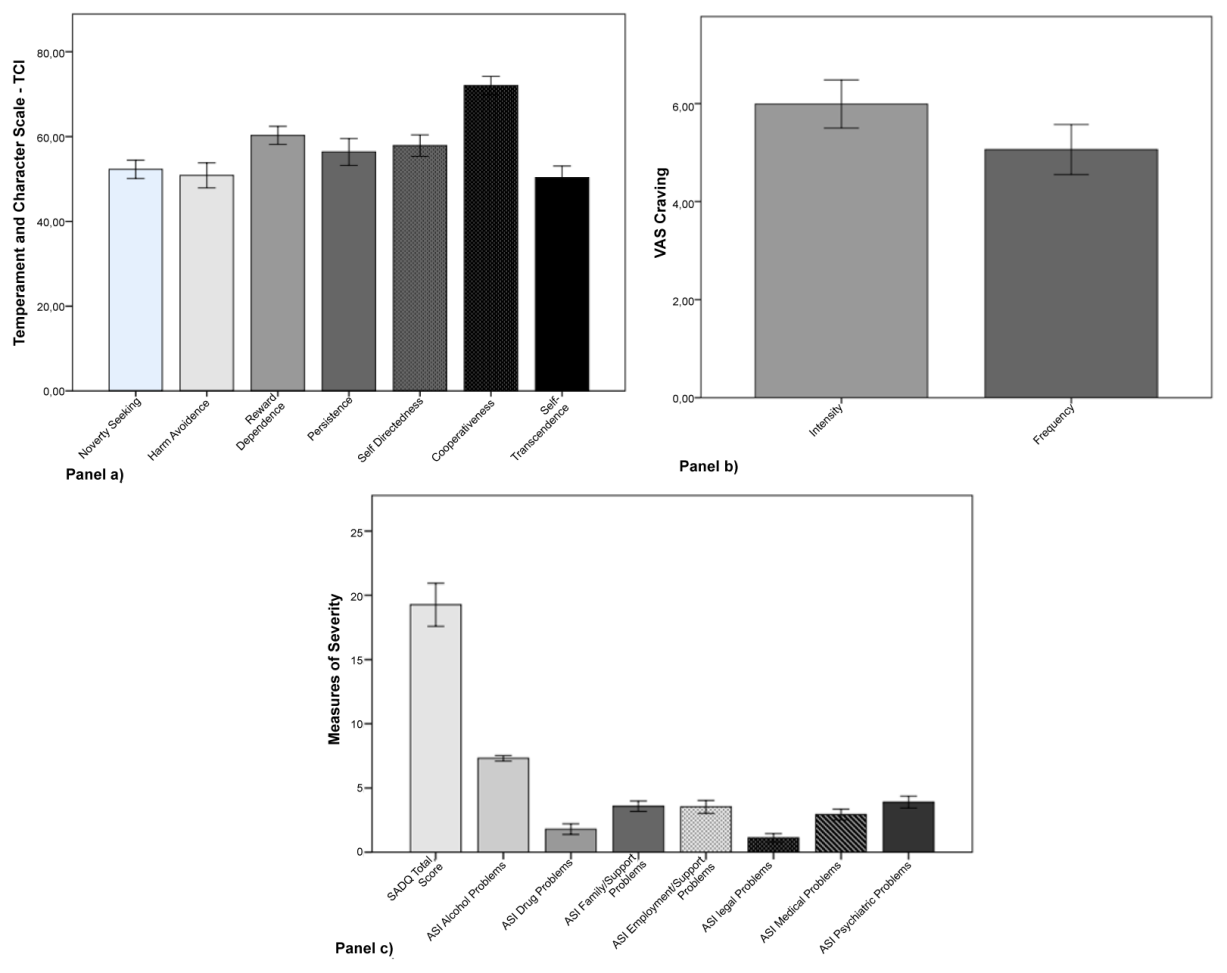
Descriptive statistics for thedifferent measures in the full sample mean and standard deviation are reported for TCI raw scores scales in panel a); VAS Craving in panel b); severity measures SADQ total score and ASI Scales in panel c).

Descriptive statistics for the different measures in the full sample are summarized in Figure 1. In our sample 81.6% of the subjects also smoked cigarettes (mean ± SD per day 17.35 ± 10.92). Since Pearson correlations were used to perform data analysis, the smoking history was not used as a covariate variable.

Utilizing TCI temperamental scales in our sample we detected a significant positive correlation between HA-scale scores and the VAS scale: increasing in HA-scale corresponds to an increase in craving perception for intensity (r=0.310; p ≤ 0.001) and frequency (r=0.246; p ≤ 0.001). Moreover, perception of dependence severity, measured with SADQ was also found to be significantly associated positively to both HA-scale (r=0.246; p ≤ 0.001) and NS-scale (r=0.224; p ≤ 0.01). As of behavioral TCI scales our data show a significant negative correlation between SD-scale and the VAS scale for craving frequency (r=−0.236; p ≤ 0.01) and intensity (r=−0.244; p ≤ 0.01). Lastly, significant negative correlations were observed between SADQ scores and SD-scale (r=− 0.295; p ≤ 0.001) and Cooperativeness (r=−0.156; p ≤ 0.01) (Table 2).

Pearson correlations between TCI temperament scales (HA, NS, RD) and dependence severity measured with ASI were not significant. (The ASI subscales reported in Table 3 are composite scores). For TCI character scales, Persistence was negative and significantly associated (r=−0.195; p=.008) with ASI linked to alcohol problems. Self-directedness was negative and significantly correlated with ASI linked to alcohol problems (r=−0.294; p ≤ 0.001), family and social problems (r=−0.349; p ≤ 0.001), employment and support problems (r=−0.220; p=0.003) and psychiatric problems (r=−0.358; p ≤ 0.001). Cooperativeness was a negative and weakly significant correlate with Legal Problems (r=−0.173; p=0.019). However, Self-Transcendence was positive and significantly correlated with Medical Problems (r=0.276; p ≤ 0.001) (Table 3).

## Discussion

While we did not find a significant cognitive impairment among our studied sample, the overall data resulting from scores of character scales seem to confirm those already observed in the scientific literature [22]. The significant differences between TCI-SADQ and TCI-ASI pattern correlations may be due to multi –factorial elements including possible psychiatric comorbidities (non-detected in this study) [55]. However, we did find that addiction severity, assessed with the SADQ, is positively correlated with both Novelty Seeking and Harm Avoidance. Finally, in the same sample, perceived craving intensity is positively correlated with high scores in the HA scale but not for the NS scale. These results are in agreement with previous work by Blum et al. [56] showing the association of the DRD2 A1 allele and schizoid/avoidance behavior.

The critical role of VTA dopamine in the regulation of affective and cognitive functions, in craving and reward has been well established [55,56]. In fact, it is well established that there is a genetic vulnerability (the DRD2 Taq1 and the DAT1 9/9 allele carriers) within the critical system of the “Brain Reward Cascade” leading to the Reward Deficiency Syndrome [57,58].

## Limitations

We are aware that significant limitations related to sample size, utilization of screened control comparison, and eventual psychiatric comorbidities may influence this research, but a possible explanation can be offered by recent scientific contributions from Koob and Volkow, with regards to the neurobiological bases of addiction [59].

## Koob-Volkow Anti-Reward Model

Koob and Volkow proposed a neurobiological pattern of addiction, composed of three phases: binge/intoxication, withdrawal/negative effect and preoccupation/anticipation. Impulsiveness often controls the early phases of this cycle, while both impulsiveness and compulsiveness characterize later phases. Neurobiological circuits seem to underlie each phase of the cycle: in the first one an important role is played by both ventral tegmental area and ventral striatum in which the enhancement of dopaminergic firing is primary; the amygdale has a key role in the second phase, while in the third one seems to be involved in a widely distributed network, formed by orbitofrontal cortex, dorsal striatum, prefrontal cortex, basolateral amygdale, hippocampus, insula and cingulated. In this circuit, the glutamatergic system appears to have an important role [59].

Transition to addiction involves neuroplastic mechanisms in all these structures; this process can begin with primary changes in the mesolimbic dopaminergic system and also changes of function in the prefrontal cortex in which seems to display an important role related to glutamatergic hyperfunction and relapse [59].

Recent findings, indeed, seem to further elucidate any of these neuroplastic mechanisms underlying an important homeostatic relationship between different brain reward neurotransmitters. For example, addictive drugs (“hijackers” of the reward homeostasis) seems to critically enhance the shift from tonic to phasic dopamine release in VTA; data seem to suggest an important VTA dopamine neuronal co-release of dopamine and glutamate; data show the critical role of “phasic dopamine” within the prefrontal cortex; evidence as pointed out by Koob & Volkow [59], suggest the possible critical role of DRD2 receptors in sustaining the dopamine neurotransmission within the prefrontal cortex and volume of brain white matter as compromised with the presence of the DRD2 A1allele. In contrast, others [60–66] and Volkow et al. [67] have correctly suggested a possible protective role of DRD2-*Taq* A2 allele in the vulnerability to addiction. Finally, data seem to suggest a possible correlation between the dysfunction of the DRD2 ***Taq A1*** allele and schizoid/avoidant behavior [56,68,69] suggesting an important link between the dopaminergic system and Harm Avoidance behavior. Taking together the data and the theories above mentioned could suggest our cohort of patients could be in a particulate stage of the course of their addiction history characterized by a hyperglutamatergic state associated with a hypodopaminergic trait and or state.

Exemplifying, alcoholics begin to drink to increase pleasure states ; then they may subsequently drink in order to relieve the discomfort of so called “mini withdrawal “ due to lack of alcohol intake. At this stage dependence has already set in and both genetic antecedents and neuro-adaptation in the entire “brain reward cascade” dominates the inner brain dysfunction of the patient [56] with particular emphasis on the complex interaction between hypodopaminergic and hyperglutaminergic interaction [55].

Our finding showing the relationship between TCI and HA in Italian alcoholics may further assist in identifying future therapeutic targets. Based on recent genetic studies from our laboratory Blum et al. [57,70] show the importance of D2 agonistic therapy in hypodopaminergic traits. We are cognizant that upstream neurotransmitter deficits in serotonergic, endorphinergic and glutamatergic systems [71] represent additional therapeutic loci. However, we propose herein, that fixing the hypodopaminergic trait/state seems parsimonious. Importantly, De Bartolomeis [72] discussed the interaction of these neurotransmitters emphasizing the role of glutamatergic hyperactivity “links to” a serotoninergic hyperfunction. In consideration of prevention of relapse, we must not only target VTA dopaminergic activity but enhance, for example, cingulate gyrus induced decision making due to poor executive functions [73]. In fact, we have already shown the benefit of a putative natural D2 agonist known as KB220Z on increasing alpha and low beta activity at the cingulate gyrus utilizing qEEG analysis promoting a significant reduction of relapse [74].

## Conclusion

In agreement with the extensive literature the TCI-based assessment of alcoholic patients enables early identification of their critical temperamental factors. Clinical utilization of these assessments allow for an optimization of treatment in terms of providing better targets that may translate to improved appropriateness and effectiveness. Our findings also suggest that clinicians should embrace these outlined hypotheses so that they could provide patients with a more comprehensive personalized analysis of AUD patients. In our opinion, discussing these newer concepts with RDS patients (AUD subset) will clinically assist the patients understanding and could suggest a functional hypothesis of neurotransmitter circuits that helps to frame the patient in his/her history of addiction.

This concept is underscored by the importance of a prescreening tool known as Genetic Addiction Risk Score (GARS) that will provide a mirror to the polymorphic gene characteristics of AUD subjects. In fact this has already been shown to be beneficial in a clinical setting [75–77].

Based on our results, we suggest required additional research comparing the TCI and other neurophysiological measures including evoked potentials such as P300 with GARS to more definitively dissect important treatment targets.

## Figures and Tables

**Table 1:** Study phases.

Study Phase	1	2	3
**Days**	From 0 to 7	8	From 9 to 13
**Interventions**	Alcohol Detoxification*	Screening**	PSYCHODIAGNOSTIC ASSESSMENT **AUD Assessment:** ASI, SADQ, VAS-C, DRIE, OCDS **Cognitive Assessment:** MMSE, WAIS-R **Character Assessment:** TCI

* According to Lejoyeux et al. [44]

** According to the inclusion/exclusion criteria

**Table 2:** Pearson correlations between TCI, VAS-Craving and SADQ

MEASURE	Visual Analogic Scale-Craving Intensity	Visual Analogic Scale-Craving Frequency	Severity of Alcohol Dependence Questionnaire
**TCI – Novelty Seeking**	.038	.029	.224**
**TCI – Harm Avoidance**	.310***	.246***	.246***
**TCI – Reward Dependence**	.035	−.007	.113
**TCI – Persistence**	−.099	−.001	−.080
**TCI – Self Directedness**	−.236**	−.244**	−.295***
**TCI – Cooperativeness**	−.102	−.141	−.156*
**TCI – Self Trascendence**	.026	.128	.078

**Note:** VAS measures in cm, SADQ total score; TCI raw scores and ASI composite scores

* p < 0.05;

** p < 0.01;

*** p < 0.001

**Table 3:** Pearson correlations between TCI and ASI.

MEASURE	ASI-Alcohol Problems	ASI-Drug Problems	ASI-Family/Social Problems	ASI-Employment/Support Problems	ASI-Legal Problems	ASI-Medical Problems	ASI-Psychiatric Problems
**TCI – Novelty Seeking**	.034	−.047	.084	.015	.142	−.051	.042
**TCI – Harm Avoidance**	.059	−.126	−.028	.031	.070	.053	−.048
**TCI – Reward Dependence**	.054	.021	−.012	.064	.009	.087	−.076
**TCI – Persistence**	−.195**	.010	−.121	−.009	.104	−.034	−.118
**TCI – Self Directedness**	−.294***	−.105	−.349***	−.220**	−.103	−.109	−.358***
**TCI Cooperativeness**	−.057	−.097	−.122	−.102	−.173*	.033	−.130
**TCI – Self Transcendence**	−.046	−.106	.042	.044	−.037	.276***	.055

* p < 0.05;

** p < 0.01;

*** p < 0.001

## Abbreviations

RDS: Reward Deficiency Syndrome
AUD: Alcohol Use Disorder
TCI: Temperament and Character Inventory
NS: Novelty Seeking
HA: Harm Avoidance
RD: Reward Dependence
P: Persistence
SD: Self-Directedness
C: Cooperativeness
ST: Self-Transcendence
ASI: Addiction Severity Index
VAS-C: Visual Analogue Scale-Craving
CIWA: Clinical Institute Withdrawal Assessment for Alcohol
GARS: Genetic Addiction Risk Score
